# Intestinal Infiltration of Chronic Lymphocytic Leukemia/Small Lymphocytic Lymphoma Found on Screening Colonoscopy

**DOI:** 10.7759/cureus.21037

**Published:** 2022-01-08

**Authors:** Kent T Aje, Ayokunle T Abegunde, Kamran Mirza

**Affiliations:** 1 Internal Medicine, Loyola University Medical Center, Maywood, USA; 2 Gastroenterology, Loyola University Medical Center, Maywood, USA; 3 Pathology and Laboratory Medicine, Loyola University Medical Center, Maywood, USA

**Keywords:** extra-nodal involvement, chronic lymphocytic leukemia (cll), small lymphocytic lymphoma (sll), gi infiltration, richter's transformation, screening colonoscopy

## Abstract

B-cell chronic lymphocytic leukemia/small lymphocytic lymphoma (CLL/SLL) is a malignancy of dysregulated lymphocytes in bone marrow and lymphatics. Extra-nodal involvement has been previously cited to affect areas like the lungs, skin, central nervous system, and kidney. Gastrointestinal (GI) involvement in patients with chronic CLL/SLL is rare. We report a unique case of CLL/SLL found incidentally in a 71-year-old asymptomatic female undergoing a screening colonoscopy. Patients with GI infiltration of CLL/SLL may be asymptomatic like the patient described in this report, or present with symptoms that may resemble inflammatory bowel disease, microscopic colitis, or colon cancer. Therefore, it is important to remain vigilant for the occurrence of other malignancies during the follow-up of CLL/SLL patients.

## Introduction

B-cell chronic lymphocytic leukemia/small lymphocytic lymphoma (CLL/SLL) occurs when dysregulated mature lymphocytes overcrowd the bone marrow, lymphoid organs, and extra-nodal lymphoid tissue [1]. CLL lymphocytes have a strong resemblance to normal mature lymphocytes and live longer than them as well, but they lack the functional properties of the normal phenotype [2]. Typically, patients can present with asymptomatic peripheral blood lymphocytosis or leukocytosis, lymphadenopathy, hepatosplenomegaly, recurrent infections, and often with autoimmune hemolytic anemia or autoimmune thrombocytopenia [2].

Extra-nodal involvement has been reported in the lungs, pleura, skin, central nervous system, and kidney [1]. Gastrointestinal (GI) involvement of CLL is very rare, and if GI involvement with symptoms were to occur, it usually occurs abruptly during Richter’s syndrome, or the transformation of CLL to an aggressive diffuse large B-cell lymphoma [2,3]. In this report, we describe a case of gastrointestinal CLL/SLL involvement found on routine screening colonoscopy. 

## Case presentation

A 71-year-old female with a past medical history of asthma, anxiety, and depression was referred for a screening colonoscopy. Her family history is significant for her father having colorectal cancer (CRC) at the age of 52 and a sister with CLL/SLL. The patient was asymptomatic and had a normal colonoscopy five years ago.

**Figure 1:**
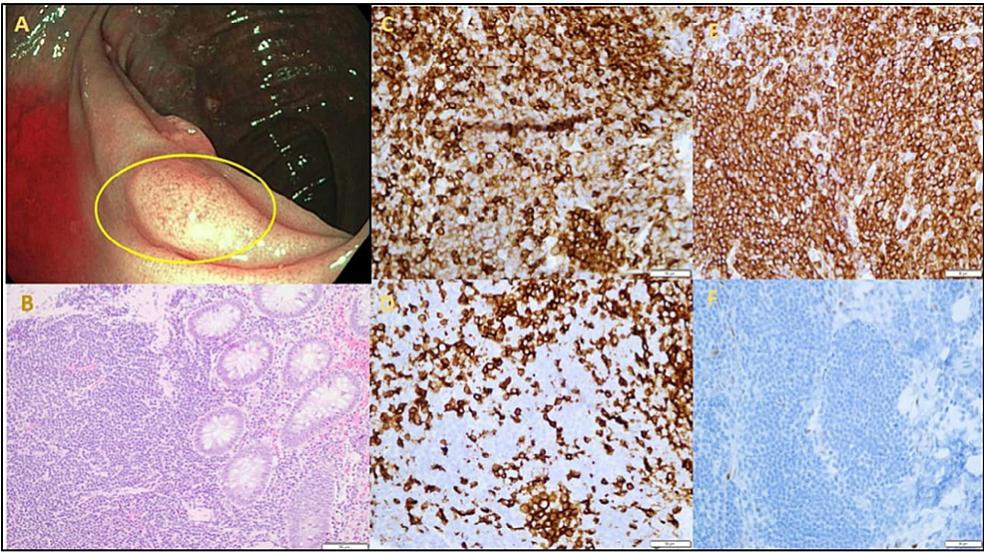
Colon Polyp A. 6 mm ascending colon polyp B. Hematoxylin and Eosin (H&E) stain a dense lymphoid infiltrate comprised of small-sized monotonous lymphocytes C. Positive CD5 stain D. Reactive T-cells highlighted by positive CD3 stain E. Positive CD20 stain F. Negative cyclin-D1 stain

Colonoscopy revealed a 6 mm sessile polyp in the ascending colon (Figure 1A), sigmoid and descending colon diverticulosis, and internal hemorrhoids. The polyp was completely resected with a cold snare. Microscopic examination of the polyp revealed colonic mucosa involved by a dense lymphoid infiltrate comprised of small, monotonous appearing lymphoma cells (Figure 1B). Immunohistochemistry demonstrated a CLL/SLL phenotype with positivity for CD20 (a B-cell marker), co-expression of dim CD5 (a T-cell marker) (Figure 1C, 1E), and absence of cyclin-D1 (a protein required in cell cycle G1/S transition) expression (Figure 1F). The lymphoma cells did not express CD10 (a surface glycoprotein), BCL-6 (a transcription repressor), or CD43 (surface glycoprotein). The background T-cells were highlighted by CD3 ( cluster of differentiation 3 protein complex) (Figure 1D) and showed equal expression of CD5. BCL-2 (apoptosis inhibitor protein) was positive in a subset of B-cells and stained background T-cells. Ki-67 (a proliferation marker protein) showed a low proliferation index (approximately 10%).

The patient was referred to hematology for further evaluation. Hematology work-up showed hemoglobin 14.7 g/dL, WBC 10 K/uL, platelets 326 K/uL, and the absolute lymphocyte count was 4.4K/mm^3^ (normal range 1.0 - 4.0 K/mm^3^). Flow cytometry of her blood confirmed the presence of a CD5 positive monotypic B-cell population expressing bright CD45 (a transmembrane protein tyrosine phosphatase), dim CD19 (transmembrane glycoprotein), dim CD20, CD5, CD23 (an integral membrane glycoprotein), and lambda light chain restriction. Flow cytometry and immunohistochemistry results can be seen in Table 1. There were no T-cell abnormalities by the markers assayed. The patient did not meet the criteria for treatment, but regular follow-up was recommended. She remains asymptomatic at 12 months of follow-up.

**Table 1:** Immunohistochemistry and Flow Cytometry Profile of CLL +/− = Dim; * = Low Proliferation (<10%); NA = Not available/Not done

Phenotype Marker	Immunohistochemistry	Flow Cytometry Expression
CD3	+	NA
CD5	+/−	+
CD10	−	NA
CD19	NA	+/−
CD23	NA	+
CD43	−	NA
CD45	NA	+
Cyclin-D1	−	NA
BCL-2	+	NA
BCL-6	−	NA
Ki-67	*	NA

## Discussion

CLL/SLL is a B-cell lymphoma characterized by the accumulation of clonal mature CD5-expressing B cells in the blood, bone marrow, lymphoid and extra-nodal tissues [4]. CLL/SLL accounts for approximately 25 to 30% of all leukemia in the U.S. Median age at diagnosis is 70 years [2,5]. Most cases of CLL/SLL are preceded by monoclonal B-cell lymphocytosis. Monoclonal B-cell lymphocytosis is a pre-leukemic state of CLL/SLL representing the asymptomatic proliferation of clonal B cells with circulating numbers <5000/μL [4]. The prevalence of monoclonal B-cell lymphocytosis increases with age, but only a few will proceed to develop CLL. The progression to CLL, at which the first oncogenic event occurs, remains unknown [4]. The differential diagnoses of CLL/SLL include Mantle Cell Lymphoma, Splenic Marginal Zone Lymphoma, Hairy Cell Leukemia, and Sezary’s Syndrome [5]. The diagnosis of typical B-cell CLL is made when an increased number of circulating lymphocytes (>4 x 10^9^/L) are monoclonal and co-express the T-cell antigen CD5 and are negative for cyclin-D1 [2,3,6]. If the primary presentation is lymphadenopathy, and a lymph node biopsy is performed, the diagnosis can be made based on morphologic findings and immunophenotype. Patients with typical B-cell CLL/SLL and no manifestations of the disease other than bone marrow involvement and lymphocytosis can be followed without specific therapy. They tend to have a median survival of 10 years.

The incidence of CLL involving the GI tract is unknown, prior autopsy results suggest that the frequency ranges from 5.7% to 13% [7]. Endoscopy may reveal normal mucosa, single polyps, multiple polypoid lesions, and focal ulceration [1,2,6]. Patients with GI infiltration of CLL/SLL may be asymptomatic like the patient described in this report, or present with persistent diarrhea, abdominal pain, and GI bleeding, mimicking inflammatory bowel disease, microscopic colitis, ischemic colitis, or colon cancer [1]. CLL/SLL sometimes occurs concomitantly with other malignant neoplasms such as melanoma, basal cell carcinoma, laryngeal carcinoma, and colon cancer [2].

Cellular and humoral immune responses are often impaired in CLL/SLL patients, and the defective immunity in these patients may play an etiological role in the reported development and rapid progression of concomitant cancers. Therefore, it is important to remain vigilant for the occurrence of other malignancies during the follow-up of CLL/SLL patients. Interestingly, the patient's sister also had CLL/SLL suggesting a familial risk for developing CLL/SLL. Genome-wide association studies have identified single nucleotide polymorphisms (SNPs) in nearly 30 locations that are associated with familial CLL/SLL [5]. The patient requires a screening colonoscopy in five years because of her family history of CRC in a first-degree relative but she does not require specific endoscopic surveillance for CLL/SLL. However, endoscopy should be considered in patients with CLL/SLL that develop new GI symptoms.

The clinical and morphologic transformation of 3 to 5% of cases of CLL to diffuse large-cell lymphoma (DLCL) is referred to as Richter’s syndrome [8]. The prognosis of Richter's syndrome is very poor, with a median survival of 5 months [8]. Parrens et al. reviewed the clinicopathology and molecular features of six cases of digestive Richter’s syndrome [7], and symptoms ranged from recurrent gastric ulcer disease, upper or lower GI bleeding, intestinal obstruction, or acute perforation [8].

Kuse and Lueb reported that the macroscopic appearance of the GI mucosa was not suitable to ascertain GI involvement in CLL [9]. Therefore microscopic examination of mucosal biopsies is crucial to diagnosing GI involvement in CLL. Management of patients with CLL ranges from monitoring to systemic chemotherapy or radiation, depending on the patient’s Rai Stage [2]. Management for Richter’s syndrome depends on whether the transformation of the CLL becomes DLBCL or Hodgkin’s Lymphoma and can include chemotherapy regimens such as R-EPOCH (rituximab, etoposide, prednisone, vincristine sulfate [Oncovin®], cyclophosphamide, and doxorubicin) or stem cell transplantation [10,11]. This report describes a case of CLL/SLL involving the GI tract without Richter's syndrome. Prior case series have suggested that lymphocytic infiltration seems to depend mainly on tumor burden and proliferation activity than on CLL staging [9]. However, in this particular case lymphocytic infiltration of the GI tract was found in a particularly low disease burden individual.

## Conclusions

Screening colonoscopy in asymptomatic patients can reveal colonic polyps or mucosal lesions infiltrated by clonal CD5-expressing B cells as the first sign of CLL/SLL. The standard management approach for asymptomatic patients with early-stage CLL/SLL is careful observation to detect disease progression and prevent complications. Diagnostic endoscopy is warranted in patients with CLL/SLL that develop unexplained GI symptoms.
